# Single‐cell RNA sequencing in oral science: Current awareness and perspectives

**DOI:** 10.1111/cpr.13287

**Published:** 2022-07-17

**Authors:** Jie Wu, Yumei Ding, Jinyu Wang, Fengyuan Lyu, Qingming Tang, Jiangyuan Song, Zhiqiang Luo, Qian Wan, Xiaoli Lan, Zhi Xu, Lili Chen

**Affiliations:** ^1^ Department of Stomatology, Union Hospital, Tongji Medical College Huazhong University of Science and Technology Wuhan China; ^2^ Guanghua School of Stomatology, Hospital of Stomatology, Guangdong Provincial Key Laboratory of Stomatology Sun Yat‐sen University Guangzhou China; ^3^ School of Stomatology, Tongji Medical College Huazhong University of Science and Technology Wuhan China; ^4^ Hubei Province Key Laboratory of Oral and Maxillofacial Development and Regeneration Wuhan China; ^5^ Center of Stomatology, Tongji Hospital, Tongji Medical College Huazhong University of Science and Technology Wuhan China; ^6^ National Engineering Research Center for Nanomedicine College of Life Science and Technolog Huazhong University of Science and Technology Wuhan China; ^7^ Hubei Key Laboratory of Natural Medicinal Chemistry and Resource Evaluation, School of Pharmacy Huazhong University of Science and Technology Wuhan China; ^8^ Institute of Brain Research Huazhong University of Science and Technology Wuhan China; ^9^ Department of Nuclear Medicine, Union Hospital, Tongji Medical College Huazhong University of Science and Technology Wuhan China; ^10^ Hubei Key Laboratory of Molecular Imaging Wuhan China

## Abstract

The emergence of single‐cell RNA sequencing enables simultaneous sequencing of thousands of cells, making the analysis of cell population heterogeneity more efficient. In recent years, single‐cell RNA sequencing has been used in the investigation of heterogeneous cell populations, cellular developmental trajectories, stochastic gene transcriptional kinetics, and gene regulatory networks, providing strong support in life science research. However, the application of single‐cell RNA sequencing in the field of oral science has not been reviewed comprehensively yet. Therefore, this paper reviews the development and application of single‐cell RNA sequencing in oral science, including fields of tissue development, teeth and jaws diseases, maxillofacial tumors, infections, etc., providing reference and prospects for using single‐cell RNA sequencing in studying the oral diseases, tissue development, and regeneration.

## Graphical abstract

The application of scRNA‐seq in oral science. scRNA‐seq has advanced insights into tissue development and oral diseases. Studies on tissue development are concerned with tooth, oral mucosa, maxillofacial alveolar bones, and salivary gland. Oral disease‐related researches include the fields of oral cancer, oral potential malignant disorders, oral and maxillofacial deformity, and infectious diseases. scRNA‐seq, single‐cell RNA sequencing.

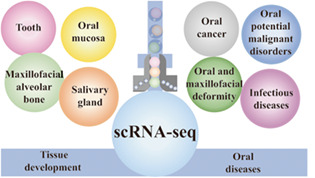

## 1 INTRODUCTION

On the analysis of mixed samples of thousands of cells, bulk RNA sequencing is prone to uncover gene expression by sequencing on average in the absence of the cellular transcriptome heterogeneity of individual cells. Traditional RNA sequencing can reveal information of dominant cell subpopulations, but the transcriptome characteristics of rare cell subpopulations cannot be shown because the results are “weighted average”.^1^ Unlike traditional RNA analysis, single‐cell RNA sequencing (scRNA‐seq) is characterized by high‐throughput and high‐resolution transcriptomic analyses of individual cells. The scRNA‐seq can be utilized in the assessment of heterogeneous cell populations, reconstruction of cellular developmental trajectories, simulation of the stochastic gene transcriptional kinetics, and inference of gene regulatory networks.^2^

Since the scRNA‐seq was first reported, scRNA‐seq has resulted in multiple pioneering studies in life science and medical research. Oral science research involves numerous areas, including oral histological structure and histogenesis, teeth and bone disease, oral maxillofacial deformity, mucosal disease, tumor disease, infectious disease, etc. Although scRNA‐seq has not been widely used in all aspects above, it is currently believed to be an indispensable tool that brings about advances in oral science. Cognizing the progress and prospects of this technique in oral science will contribute to the progress in oral science. Herein, current progress and underlying prospect for scRNA‐seq in researches of oral science will be reviewed and discussed in this review.

## 2 THE BRIEF HISTORY OF SCRNA‐SEQ DEVELOPMENT

**FIGURE 1:**
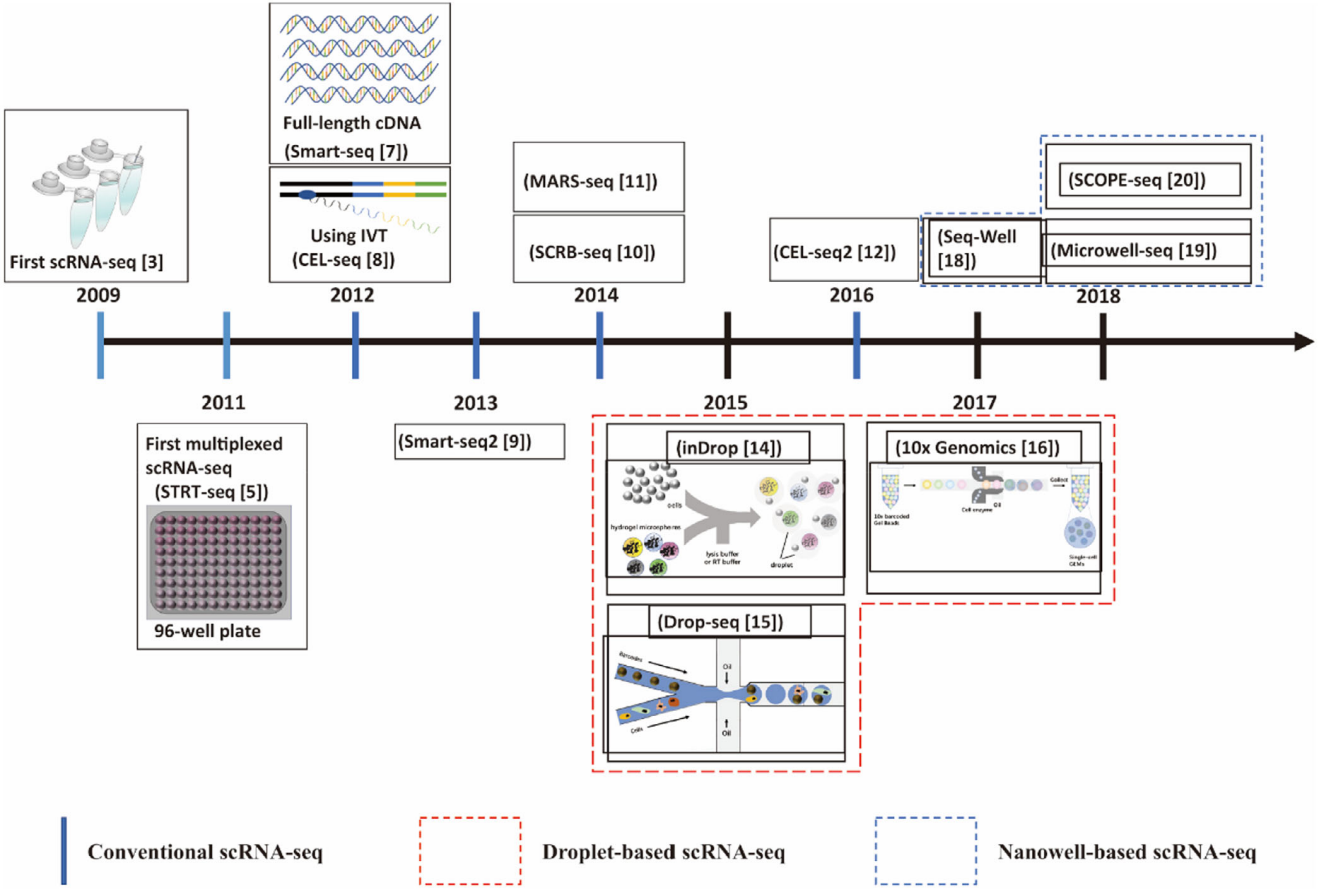
The brief history of scRNA‐seq development. The three categories, conventional scRNA‐seq, droplet‐based scRNA‐seq and nanowell‐based scRNA‐seq, are classified according to Choi et al.^13^

Traditional RNA sequencing requires microgram amounts of total RNA for analysis. Therefore, scRNA‐seq was first reported by Tang et al.^3^ in 2009 to tackle the problem that the samples obtainable were too scant to implement transcriptome analysis, which preferably can analyze the samples at single‐cell level. In Tang et al.’s protocol, scRNA‐seq dataset relied on manual manipulation of individual cells, thus it was unable to achieve multiplexing.^4^ Single‐cell tagged reverse transcription sequencing (STRT‐seq), the first multiplexed scRNA‐seq, was put forward by Islam et al.^5^ in 2011. STRT‐seq permits the introduction of a barcode for multiplexing, which allows simultaneous amplification of cDNAs from tens of thousands of cells, thereby reducing pre‐processing of cells and reducing cost.^6^ Nonetheless, only the 5′ end of each cDNA is quantified by STRT‐seq. Subsequently, switching mechanism at the 5′ end of RNA template sequencing (Smart‐seq), developed by Ramsköld et al.^7^ in 2012, has significantly improved full‐length coverage of all transcripts longer than 1 kb reverse‐transcribed using Moloney Murine Leukemia Virus Reverse Transcriptase (MMLV‐RT). Distinct from the PCR‐based protocols aforementioned, linear in vitro transcription (IVT) amplification was recommended in Cell Expression by Linear amplification and sequencing (CEL‐seq) proposed by Hashimshony et al.^8^ It is demonstrated that CEL‐seq yielded more repeatable, sensitive and linear‐amplified results compared with the PCR‐based protocols. Nevertheless, in addition to 3′ bias, CEL‐seq has the disadvantage of low sensitivity to lowly expressed transcripts.^8^ In addition to the aforementioned strategies, conventional scRNA‐seq technologies include Smart‐seq2,^9^ Single Cell RNA Barcoding, and Sequencing (SCRB‐seq),^10^ Massively Parallel Single‐Cell RNA‐Sequencing (MARS‐Seq),^11^ CEL‐seq2^12^ et al. They have made progress in automation and reduction of cost and reaction volume. However, they remain labor‐intensive and time‐consuming.^13^ Some droplet‐based scRNA‐seq technologies, such as inDrop (indexing droplets) RNA sequencing,^14^ Drop‐seq,^15^ and 10× Genomics,^16^ have been invented to make the sequencing of a large number of cells more high‐throughput. Hydrogel microspheres are utilized to introduce barcoded DNA oligonucleotides to label each cell in inDrop, and reactions, including cell lysis, marking cells by barcodes, and cDNA synthesis, are performed in droplets. Successively, the cDNA is amplified by IVT. Drop‐seq, unlike inDrop, uses barcoded beads to introduce oligonucleotides and PCR to amplify cDNA.^15^ Gel bead in Emulsion (GEM), which significantly elevates cell capture efficiency, is the pivotal technology of 10× Genomics and 10× barcodes are exploited to further improve the throughput.^16^ Summary of these three most prevalent droplet‐based scRNA‐seq technologies is presented in Table 1. Apart from droplet‐based scRNA‐seq technologies, nanowell‐based scRNA‐seq technologies, such as Seq‐Well,^18^ Microwell‐seq,^19^ single cell optical phenotyping and expression sequencing (SCOPE‐seq)^20^ et al., have been developed recently and endowed with the advantages of more simplified preparation of cells, improved optimization of optical imaging, fewer experiment reagents, and lower reaction volume.^13^ The history of scRNA‐seq development was summarized in Figure 1.

**TABLE 1:** The comparison of inDrop, Drop‐seq and 10× Genomics

	inDrop	Drop‐seq	10× Genomics
Introducing the oligonucleotides	Barcoded hydrogel microspheres	Barcoded beads	Barcoded gel beads
Barcode capacity	~150 000	~16 000 000	~750 000
Amplification method	IVT	PCR	PCR
Cell capture efficiency^17^	~25%	~2%	~50%
High throughout	+	++	+++

## 3 RE‐ANALYSIS OF ORAL HISTOLOGICAL STRUCTURE AND HISTOGENESIS USING SCRNA‐SEQ

The profiles of transcriptome, epigenetics, and niche endow each single cell with unique characteristics. Through scRNA‐seq, researchers can determine expression profiles of oral histology at single‐cell resolution, which can clarify cell subpopulations, record signature genes, track variable cellular development trajectory and recognize intercellular crosstalk.

### 3.1 Tooth histogenesis

**FIGURE 2:**
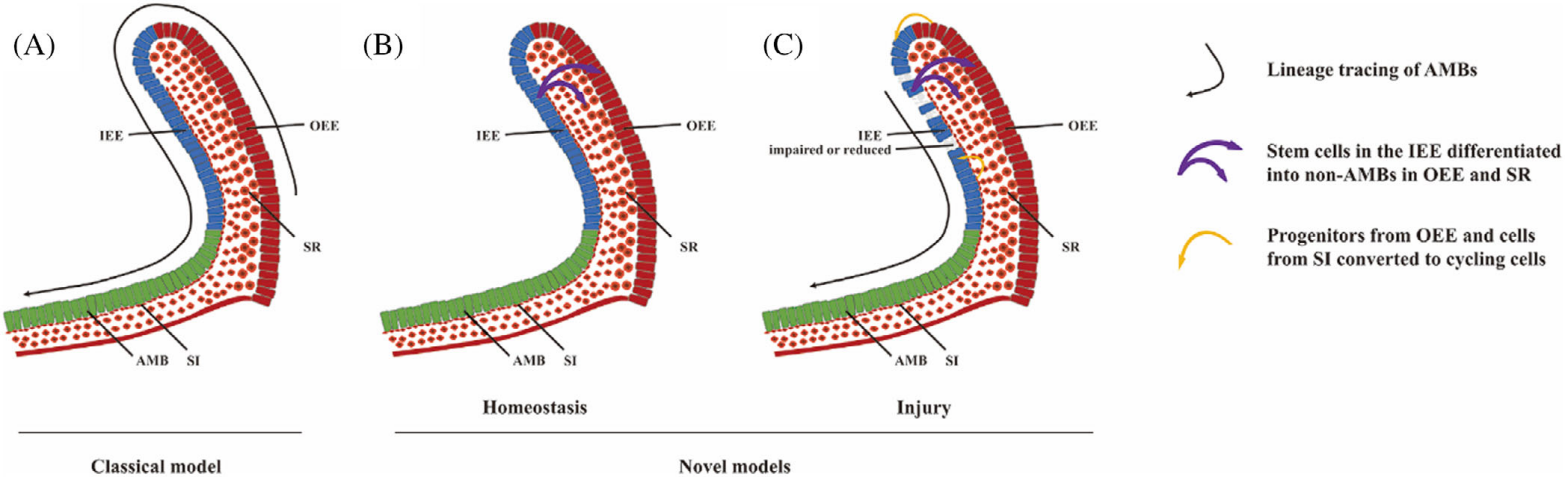
Reanalysis of location and lineage differentiation of dental epithelial stem cells from mouse incisors by scRNA‐seq^22^, ^23^ (A) Progenitor cells located in the proximal portion of the outer enamel OEE (red) or SR (orange) of the laCL yielded the IEE cells (blue), and finally contributed to AMBs(green) according to classical model.^23^ (B) During homeostasis, the stem cells residing in the IEE differentiated into AMBs, and another small population of the stem cells differentiated into non‐AMB epithelial cells in OEE and SR according to novel model via scRNA‐seq.^22^ (C) When IEE cells were impaired and reduced during injury caused by 5‐fluorouracil, the progenitor cells from OEE and SI converted to cycling cells, differentiating into AMBs for tooth repair according to novel model via scRNA‐seq.^22^ laCL: labial Cervical Loop; AMBs: Ameloblasts; SI: Stratum Intermedium; SR: Stellate Reticulum; IEE: Inner Enamel Epithelium; OEE: Outer Enamel Epithelium.

The current restoration of tooth defects and deletions relies on synthetic materials without biological activity. A comprehensive understanding of the cellular and molecular mechanisms that regulate tooth structure and developmental processes will benefit the development of tooth regeneration engineering.^21^ The scRNA‐seq can help re‐examine the oral structure from the perspective of single‐cell resolution, facilitating the construction of oral structure profile. The use of single‐cell sequencing has been applied for studies on teeth, whereas the focus differs. The scRNA‐seq adopted in research from Sharir et al. was aimed to uncover the characteristics, locations, and function of dental stem cell.^22^ Previously, the classical model of dental epithelial stem cells (DESCs) from mouse incisors suggests that the progenitor cells in the labial cervical loop (laCL) are in the vicinity to the outer enamel epithelium (OEE) or stellate reticulum (SR), which generate the transit‐proliferating inner enamel epithelium (IEE) cells and terminally give rise to all the epithelial progeny cells (Figure 2A).^23^ In contrast, the model from Sharir et al. pointed out that during homeostasis the stem cells residing in the IEE differentiated into ameloblasts and a small population of non‐ameloblast epithelial cells in OEE and SR (Figure 2B). Upon injury of proliferative IEE cells, the progenitors from OEE and cells from stratum intermedium (SI) entered cycling phase, differentiating into ameloblasts during the recovery state (Figure 2C). These results were further validated by RNAscope or immunofluorescence staining.^22^ With scRNA‐seq, another research group discovered novel cell types and marker genes of dental epithelial cells in mice. Dental epithelial cells from incisors of postnatal day 7 mice were reclassified, identifying two novel subpopulations of ameloblasts in secretory stage and some previously unknown dental epithelial cell marker genes. Pseudotime analysis suggested one novel subpopulation of ameloblasts (Dentin Sialophosphoprotein [Dspp] +) differentiates into the other one (Ameloblastin [Ambn] +).^24^ Krivanek et al. applied scRNA‐seq to explore the novel histology hierarchy of self‐recycling mouse incisors as well.^25^ For example, Ryanodine Receptor 2 (RYR2) + ameloblast subpopulation and Thrombomodulin (THBD) + SI subpopulation, unrecognized cell subpopulations previously, were detected by scRNA‐seq. In addition, the research analyzed the similarities and differences between growing mouse incisor and non‐growing mouse molar and evaluated how similar mouse tooth model and human tooth biology are.^25^ Fresia et al.^26^ discussed essential similarities and differences between the three studies above. For example, a more complex hierarchy of mouse incisor epithelial cell populations was reported in the studies from Sharir et al.^22^ and Krivanek et al.^25^ However, possibly owing to the more immature mice model sampled, fewer cell clusters were reported by Chiba et al.^24^ It was likely due to variations in the analysis tools and parameters adopted as well.^26^ Meanwhile, all three studies discovered a great amount of previously unrecognized markers in the nonameloblast counterpart.^26^

The scRNA‐seq can help map the differentially expressed genes (DEGs) to their spatial locations from single cell perspective.^27^ Keratin 15 (Krt15) in the OEE as well as claudin‐10 (Cldn10) in the SI was identified in all three scRNA‐seq studies. However, not all marker genes discovered by scRNA‐seq were functional and critical genes in tooth histogenesis. The cap analysis of gene expression sequence (CAGE‐seq) can identify the preferentially expressed genes in a particular organ without transcript length bias, thus helping determine if the genes identified by scRNA‐seq are preferentially expressed in tooth.^28^ Combined scRNA‐seq with CAGE‐seq, Cldn10 was found to be a novel SI marker,^29^ as well as FXYD domain‐containing ion transport regulator 4 (Fxyd4) and Dspp to be unrecognized ameloblast markers.^28^ The mouse incisor serves as a major model to study tooth development. The in‐depth exploration of the model by scRNA‐seq updates our knowledge of the development and maintenance of tooth.

The comparison in scRNA‐seq expression profile of mouse and human teeth revealed the differences between them. Some limitations may exist when using mouse teeth as model for human teeth.^25^ Therefore, it is necessary to picture the comprehensive expression profiles of human teeth further by scRNA‐seq. The scRNA‐seq was used to discover the cellular heterogeneity and molecular signatures in human pulp.^30^, ^31^, ^32^, ^33^ From dynamics and differentiation trajectories analysis, endothelial cells exhibit the most dynamic behavior, while only minor differentiation trajectories are found in most dental pulp cell populations.^31^ According to the ligand‐receptor pair analysis among cell populations, the pulp cells communicated the most with other cell types, while T cells communicated the least.^31^

Owing to its differentiation potential, human dental pulp stem cell (hDPSC) is promising for tooth regeneration engineering.^34^ scRNA‐seq can decipher some unrecognized traits of these stem cells. Based on the scRNA‐seq data, Notch3 was found as a marker for hDPSC, which was also identified by lineage tracing in mouse tooth injury model.^35^ Furthermore, from scRNA‐seq analysis, the fate of hDPSC was possibly determined by the microenvironment it resided.^30^, ^32^ It is noteworthy that the cell population analysis on scRNA‐seq data showed that monolayer culture of hDPSCs was significantly different from freshly isolated hDPSCs in cellular composition.^36^ Hence, it may affect the application of hDPSC in tooth regeneration engineering.^36^

Overall, based on scRNA‐seq technique, these studies have established a more concrete profile of the cellular hierarchy, identity, position, and function during tooth histogenesis. The scRNA‐seq data on teeth improve the interpretation of the cell biology and molecular mechanisms about the histogenesis of teeth,^26^ which helps the development of teeth regeneration engineering.

### 3.2 Oral mucosa

Oral mucosa is an essential barrier for human, and is constantly exposed to commensal microbiota and airborne antigens. Oral mucosal also withstands dietary antigens and frequent damage from mastication.^37^ The scRNA‐seq can contribute to unraveling the mystery of the unique barrier function of oral mucosa. The scRNA‐seq has been used to analyze oral mucosa samples taken from different tissue layers and sites. A profile of cellular heterogeneity and organization of the basal layer was established in research from Jones et al. via scRNA‐seq. It was detected that the basal epithelium consists of progenitor and post‐mitotic cells at different stages.^38^ Recently, the first human gingival cell atlas has been set up using scRNA‐seq, uncovering the heterogeneity within major gingiva cell populations.^39^ Another study by Williams et al. obtained scRNA‐seq data from biopsies of the buccal and gingival mucosa, which profiled the cell hierarchy and molecular attributes of the oral mucosa.^40^ A specific keratinocyte subpopulation was merely found in the gingiva, but not in the buccal mucosa. The DEGs analysis showed its top expressed genes included antimicrobial and inflammatory factors, and leukocyte chemotaxis pathway was identified as a top pathway, which may act through recruitment of neutrophils.^40^ On the other hand, periodontal tissue was collected for scRNA‐seq analysis as well. The molecular signatures of mesenchymal stem cells (MSCs) in periodontal tissue were discovered to be similar to that in dental pulp.^31^ The spatial distribution of Peroxiredoxin 1 (PRX1) + cells, high proportion cells in the periodontal tissue of the third molar, was confirmed based on combination of scRNA‐seq and immunofluorescence staining.^41^ Overall, the analysis of cell populations and gene expressions of oral mucosa by scRNA‐seq provides the future research with split‐new perspective.

### 3.3 Maxillofacial alveolar bones

The 10× Genomics technology was used to profile single‐cell transcriptome of mouse mandibular alveolar bone^42^ and the study discovered that immune cells predominated the mandibular microenvironment. The scRNA‐seq data of long bone (femur) were compared with that of alveolar bone (mandible) to highlight the immune cell profiles in alveolar bone. Alveolar bone marrow has a higher proportion of activated macrophages, which are the major population secreting the Oncostatin M (Osm), compared with long bone marrow. The cellular ligand/receptor pairing‐based analysis using CellPhoneDB2 showed that macrophage cluster has the most interactions with MSCs.^42^ Differences in myeloid progenitor cell populations in alveolar bone and long bone were compared. There were significantly fewer myeloid‐derived suppressor cells (MDSCs) in alveolar bone but they exhibited higher immunosuppressive activity.^43^ The immune cell atlas of alveolar bone revealed by scRNA‐seq laid a solid foundation for in‐depth research on how alveolar bone plays an essential role in orthodontic tooth movement, and reacts to inflammatory diseases, occlusal stress stimulation etc. For example, it is believed that the overreaction of the innate and acquired immune systems induced by dental plaque deteriorates periodontal tissues in periodontal lesions.^44^ The orthodontic tooth movement is characterized by alveolar bone resorption, triggered by periodontal immunoreaction upon mechanical stimulus.^45^ Therefore, the scRNA‐seq atlas of the immune microenvironment in the alveolar bone may be beneficial for understanding the immune responses during physiologic and pathological bone remodeling from a new perspective.

Modification of the mandibular arch, the most rostral element of the pharyngeal arches, facilitates skeletal structure of vertebrate jaws.^46^ The formation of jaws depends on neural crest‐derived mesenchyme developed along the proximal‐distal as well as the oral‐aboral axis.^47^ The detailed gene expression at single cell resolution provided by scRNA‐seq has made it possible to explore the molecular atlases that mediate histogenesis and organogenesis.^48^, ^49^ Using embryonic day 10.5 mouse mandibular arch, cluster analysis by scRNA‐seq with tSNE distributed single cell to their subpopulations along rostral‐caudal, oral‐aboral, and proximal‐distal axis of mandibular arch.^50^ Two subpopulations highly expressed Heart and Neural Crest Derivatives Expressed 2 (Hand2) which is a marker of the mandibular arch mesenchymal distal domain in the mouse embryo after 10.5 days. One of these two subpopulations was characterized by high expression of Sonic Hedgehog (Shh) signaling. The other was endowed with top expression of Bone Morphogenetic Protein 4 (BMP4) signaling genes. The in‐situ hybridization and immunofluorescent staining confirmed the activation of Shh signaling pathway complemented the activation of BMP4 signaling pathway along the oral‐aboral axis in mandibular arch of mouse embryo. Histological analysis indicated that Shh signaling in the neural crest‐derived mandibular mesenchyme is essential for inducing tongue formation and preventing osteogenic differentiation of the oral side of the developing mandible.^50^ In a word, the molecular map created by scRNA‐seq assists in understanding the organogenesis of jaws and promotes the research on the formation of craniomaxillofacial bones.

### 3.4 Salivary gland

Organogenesis is accompanied by complicated network of a variety of different cell populations. The gene expression of major progenitor cell population collected by bulk RNA sequencing cannot unearth the critical signals that drive the specific differentiation. It's necessary to depict the gene expressions in the early states that determine the differential fates of a single cell by scRNA‐seq.^51^ The scRNA‐seq data of young adult mouse submandibular gland epithelium was generated to trace the epithelial cell trajectory of development. Focused on basal and myoepithelial cell subpopulations, the study discovered that cells with p63 expression served as multipotent stem cells. Not only during embryogenesis but also in adult state can all epithelial cell type be derived from these cell populations. Nevertheless, smooth muscle actin (SMA) + myoepithelial cells, multipotent cells as well, merely contributed to the myoepithelial and ductal cell types in adult state.^52^ The research from Oyelakin et al.^53^ also reveals the complexity of cellular lineages in the parotid gland by scRNA‐seq and detects a novel cell subpopulation expressing almost all the ductal, acinar, and epithelial specific genes. This unrecognized cell cluster may be a cell type with diverse differential fates,^53^ which can develop into multiple cell lineages, and similar cell populations with mix‐lineage were previously reported in the mammary gland,^54^ and hematopoietic system.^55^ In addition, unique cellular behaviors and molecular markers for the bud initiation stage have been probed by scRNA‐seq. The first scRNA‐seq study of bud initiation focused on the transcriptome of mesenchymal cell populations.^56^ Interestingly, a muscle cell population, prominent in the parotid of embryonic day 12 mice, was neither myoepithelial nor vascular smooth muscle, and expressed genes that are responsible for skeletal muscle differentiation and function. A neuron cluster, originated from submandibular gland but not in parotid, co‐expressed Tubulin Beta 3 Class III (Tubb3) and noradrenergic neuron differentiation marker genes such as Hand2. Analysis on differential gene expression and localization of neuronal and muscle gene markers in salivary glands deciphered the relationship of salivary epithelia and their surrounding neuronal and muscle precursors in embryonic submandibular versus parotid glands.^56^ In addition, the differentiation programming with cellular heterogeneity in key developmental events was identified by single‐cell transcriptome of mouse submandibular glands. Combined scRNA‐seq with lineage tracing was used to identify transcription factors likely involved in acinar and ductal differentiation and characterize subsets of discrete cell types in anatomically defined glandular compartments.^57^ In addition to constructing transcriptome maps of mouse salivary glands, scRNA‐seq also created transcriptome maps of human parotid gland^58^ and minor salivary gland^59^ from single‐cell perspective. The scRNA‐seq data of human parotid gland was compared with that of human minor salivary gland, other human digestive glands, and adult mice parotid gland.^58^ As a result of scRNA‐seq analysis, a comprehensive atlas of the heterogeneity of cell populations and expression profiles in salivary glands has been compiled.

## 4 IN‐DEPTH STUDY OF ORAL DISEASES USING SCRNA‐SEQ

### 4.1 Oral cancer

#### 4.1.1 Special gene profiles

Based on analysis of expression profiles for hundreds of head and neck squamous cell carcinomas (HNSCC) tumors, HNSCC was classified by The Cancer Genome Atlas (TCGA) study into four subtypes: basal, mesenchymal, classical, and atypical.^60^ Nonetheless, the bulk data cannot reveal the intra‐tumoral heterogeneity. The scRNA‐seq can provide an alternative solution. The expression profiles of malignant, stromal, and immune cells in oral squamous cell carcinoma (OSCC), an integral part of HNSCC, were depicted by scRNA‐seq, to evaluate their corresponding subtype expression signatures.^61^ It is indicated that the malignant cells were mapped to three of OSCC subtypes (classical, atypical, basal). None of malignant cells were able to map the mesenchymal subclass, but the stromal cell populations were able to map to it. Therefore, the mesenchymal subtype may reflect malignant‐basal subcategory containing lots of stromal cells, and OSCC tumors may be reclassified into malignant‐basal, classical, and atypical subtypes.^61^ Classification of OSCC is of clinical significance, providing guidance on therapy selection, treatment prognosis, and tumor progression monitoring. More accurate classification of tumor can be obtained by the high resolution of scRNA‐seq that takes intra‐tumor and inter‐tumor heterogeneity into consideration.

C‐X‐C Motif Chemokine Ligand 14 (CXCL14), a highly conserved and homeostatic chemokine, is in charge of immune cell recruitment and maturation, as well as affecting epithelial cell motility, and thus contributes to the establishment of immune surveillance within normal epithelial layers.^62^ The role of CXCL14 in growth inhibition and apoptosis of tumor cells, and tumor‐infiltrating lymphocytes (TIL) has been validated in human papillomavirus (HPV)‐positive head and neck cancers.^63^ Nevertheless, the detailed mechanism of CXCL14 in HPV‐negative OSCC has not yet been discovered.^64^ Moreover, the transcriptome of a critical subpopulations of cancer cells may be omitted by bulk RNA sequencing because the data produced reflect the average expression of all cells. Herein, the scRNA‐seq was used to overcome these shortages. scRNA‐seq data revealed that malignant cells in the lymph nodes (LNs) had significantly down‐regulated CXCL14 expression compared to those in the primary tumor. In vitro and in vivo experiments show that higher CXCL14 expression in tumor cells is associated with decreased tumor growth and increased TIL. In the scRNA‐seq data of human patient samples, CXCL14 expression was only associated with TIL in malignant cells and not found in other non‐malignant cells, suggesting that CXCL14 expression is cell‐specific. The overall CXCL14 expression was not found related with TIL according to the TCGA cohort, indicating the weakness of bulk RNA sequencing and the necessity of applying scRNA‐seq for gene expression in cancer research.^64^ On one hand, based on the expression level of CXCL14, prognosis of the OSCC patients can be predicted. On the other hand, CXCL14 may be a prospective target for cancer treatment.^65^ It was reported that CXCL14 could be used to screen cetuximab‐responsive patients.^66^ These results suggest that scRNA‐seq can be used to detect molecular targets of oral cancer that are difficult to be found or ignored by conventional sequencing technology.

The epithelial‐to‐mesenchymal transition (EMT) program converts epithelial cells to cells that entered mesenchymal cell states, expressing the classical transcription factors such Snail Family Transcriptional Repressor 1/2 (SNAI1/2), Twist Family BHLH Transcription Factor (TWIST) etc., which enables carcinoma cells to complete multi‐step process of the invasive and metastatic cascade.^67^, ^68^ The mechanisms and significance of EMT in human epithelial tumors remain opaque. Comprehensive information on gene expression profiles of individual tumor cells can be provided by scRNA‐seq, offering a novel perspective on the process of primary tumor and metastases.^69^ The scRNA‐seq was utilized to examine the extracellular matrix program of oral cancer, and partial epithelial‐to‐mesenchymal transition (p‐EMT) program in malignant cells was identified, which was revealed by expression of epithelial markers (e.g., multiple cyto‐keratins) but not EMT‐specific TFs, with the exception of SNAI2.^61^ Combined scRNA‐seq data with immunohistochemistry, it was also demonstrated that the location of cells with p‐EMT program was at the front edge of the tumor and adjacent to cancer‐associated fibroblasts (CAFs), inferring that the p‐EMT program played potential roles in tumor invasion. In addition, higher p‐EMT scores was associated with nodal metastasis and adverse pathologic properties.^61^, ^70^ It was proposed that p‐EMT program provides cancer cells with multi‐functionality and multi‐identity so that these cells have the capacity to adapt various microenvironments.^71^ The strong plasticity of cancers was attributed to their adaptability, leading to the difficulty and complexity for cancer treatments.^72^ Also, p‐EMT program has clinical significance in guidance for the treatment because of its promising prediction of nodal metastases, lymphovascular invasion as well as extranodal extension in OSCC patients.^61^ The discovery of p‐EMT by scRNA‐seq, which is likely to be more aggressive intrinsically and cannot be detected by current histopathologic technology, may point out the requirement for adjuvant therapy without any pathological manifestation.^73^ In the future, by applying scRNA‐seq, it is of importance to determine whether the cancer cell in the leading edge of tumor is undergoing p‐EMT program.

Furthermore, the scRNA‐seq data could serve as comprehensive resources for discovery of new cancer targets and mechanisms. For example, the scRNA‐seq data^61^ demonstrated that CDC28 Protein Kinase Regulatory Subunit 2 (CKS2) was positively correlated with Cyclin B1 (CCNB1) and Cyclin A2 (CCNA2) expression in TSCC tumor cells, which suggests that CKS2 may act as an important regulator of the G2/M phase transition.^74^ In addition, the expression profiles of prognosis‐related S100 protein family members in human HPV‐negative OSCC were evaluated using the same scRNA‐seq data. Combined with the bulk RNA‐seq data, S100 Calcium Binding Protein A13 (S100A13) was detected as a significant differentially expressed gene. Upregulation of S100A13 was related to decreased OSCC sensitivity to cisplatin.^75^ Moreover, DEG analysis on scRNA‐seq data demonstrated that upregulation of pro‐coagulant (Coagulation Factor III [F3]) and pro‐fibrinolytic (Plasminogen Activator, Urokinase [PLAU]) genes coexisted in an OSCC subpopulation.^76^ Accumulating novel potential therapeutic targets have been revealed by scRNA‐seq resources. More experiments in vitro and vivo should be carried out to ascertain their intrinsic effect on oncogenic progression.

#### 4.1.2 Tumor microenvironment (TME) discovery using scRNA‐seq

TME, consisting of non‐malignant cells and extra cellular matrix (ECM), interacts with tumor in an intricate way. The non‐malignant cells of tumor include various cell populations, such as CAFs, endothelial cells, pericytes, immune inflammatory cells et al.^77^ The scRNA‐seq allows identification of heterogeneity and hierarchy of the non‐malignant cell populations. It is reported that these non‐malignant cells were not specific to the HNSCC patients, in contrast to malignant cells,^61^ indicating that a common pathogenesis was possessed by these TME cells. Therefore, a similar drug therapeutic protocol may be suitable to these patients.

The fibroblasts in TME of HNSCC were separated into two main subtypes‐myofibroblasts (MFBs) and CAFs, and a third minor subtype‐resting fibroblasts based on scRNA‐seq studies.^61^ Some OSCC cases showed a network pattern of arrangement of myofibroblasts which was proved to be responsible for more invasive behavior of the tumor.^78^ CAFs have been shown to play a critical part in TME alterations that promote tumorigenesis and progression.^79^ The p‐EMT malignant subpopulations in the leading margin of tumor was adjacent to CAFs. Based on the prediction of receptor‐ligand interactions inferred by single cell expression, p‐EMT malignant cells highly expressed the receptor transforming growth factor β‐induced (TGFBI), and CAFs highly expressed TGFB,^61^ highlighting that CAFs‐malignant cell cross‐talking is likely to contribute to invasiveness and metastasis of OSCC. The release of TGF‐β from malignant cells may induce secretion of tumor‐promoting chemokines by CAFs, and the tumor‐promoting chemokines have an effect back on cancer cells, facilitating cancer invasiveness.^80^ The roles of CAFs in driving cancer cell proliferation, maintaining cancer stemness, T cell infiltration, promoting resistance to chemotherapy et al. have been summarized previously.^81^

Some rare immune cell subpopulations and their representative gene expression patterns have also been discovered by scRNA‐seq. The regulatory CD4+ T cells and exhausted CD8+ T cells in OSCC have been detected using scRNA‐seq. Chen et al.^82^ found programmed death protein 1 (PD‐1) and cytotoxic T lymphocyte‐associated protein 4 (CTLA4), the immunosuppressive checkpoints, were identified as marker genes in exhausted CD8+ T cells, while forkhead box P3 (FOXP3) and CTLA4 were reported as marker genes of regulatory CD4+ T cells. Except the immunosuppressive checkpoints, the CD4+ regulatory T cells showed high expression of co‐stimulatory genes such as CD28 and Inducible T Cell Costimulator (ICOS).^82^ The scRNA‐seq makes for characterizing the T cell subpopulations of OSCC and their gene expression profiles, advancing the checkpoint immunotherapy in OSCC. The high resolution of scRNA‐seq extracted a Transcription Factor 7 (TCF7+) subpopulation in OSCC.^83^ The DEG analysis of scRNA‐seq data indicated these cells expressed high levels of tertiary lymphoid structures (TLS)‐related genes and low levels of immune checkpoint molecules such as CTLA4 and Lymphocyte Activating 3 (LAG3), which was associated with good prognosis.^83^ Immunosuppressive drugs have achieved certain clinical effects, but not all HNSCC patients are sensitive to them. For example, results of clinical trials indicated that the overall response rate to the programmed death protein 1 (PD‐1)‐targeted drug was under 20%.^84^ Identification of immune cell subsets and markers associated with immune evasion will benefit the development on novel immunosuppressive drugs.

The profiles of tumor immune microenvironment (TIME) caused by different etiologies can be also compared by scRNA‐seq. OSCC can be induced by environmental carcinogens and several high‐risk types of HPV, thus which can be classified into HPV− and HPV+ OSCC respectively.^85^, ^86^ The scRNA‐seq transcriptional profiles of the immune cells from HPV− and HPV+ OSCC were compared. Lineage tracing revealed the developmental trajectory of CD4+ and CD8+ T cells. The scRNA‐seq and immunofluorescence analysis uncovered that in both HPV– and HPV+ TILs, ligand/receptor interactions between B cells and Tconv CD4+ cells were found, but interactions between germinal center B cells and T follicular helper cells were only found in HPV+ TILs.^85^ The TIME comparison between the mutation‐ and virus‐driven OSCCs based on scRNA‐seq data promotes related research on how HPV infection causes HPV‐positive OSCCs.

#### 4.1.3 CSCs dissection using scRNA‐seq

With high resolution, scRNA‐seq has competence to dissect cancer stem cells (CSCs), a small cell population with specific gene signatures.^87^ An increasing number of experimental and clinical data supports that a minor cluster of CSCs that can self‐renew may contribute to tumor recurrence and metastasis. Due to chemo‐resistance and radio‐resistance of CSCs, the predictability of treatment efficacy based on macroscopic tumor response is poor.^88^ CSCs have been dissected by scRNA‐seq in a few types of cancers.^87^, ^89^, ^90^, ^91^ Using known markers of CSCs,^92^ the presence of CSCs was validated in scRNA‐Seq data.^93^ Mediator Complex Subunit 28 (MED28) was detected as DEG of OSCC by scRNA‐seq, upregulation of which was reported to increase the OSCC stem‐cell like activity.^94^ Seventy‐four genes with significantly higher expression in breast cancer stem cells than in non‐CSCs were identified by scRNA‐seq, many of which were not markers of breast cancer, consistent with the idea that there is a shared expression profile among the CSCs from different cancers.^90^ The identification of tumor stem cells by scRNA‐seq may reveal the underlying mechanisms of tumor therapy resistance and tumor recurrence.

**FIGURE 3:**
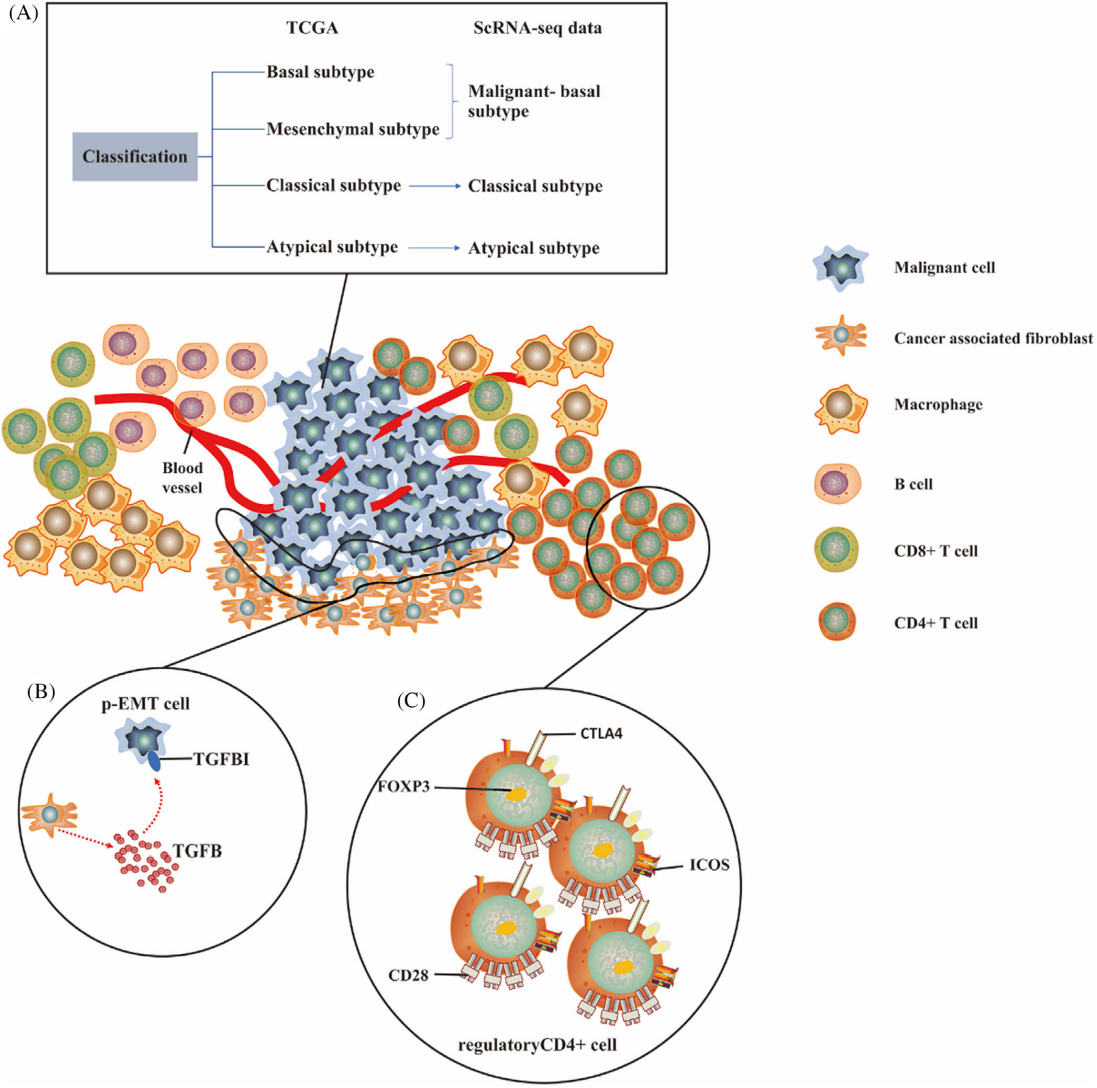
Illustration for key findings of OSCC through by scRNA‐seq. (A) The heterogeneity of malignant cells revealed by scRNA‐seq. For example, OSCC was reclassified into malignant ‐basal, classical, and atypical subtype according to expression profiles of malignant cells.^61^ (B) Invasiveness and metastasis of OSCC revealed by scRNA‐seq. For example, p‐EMT cells, located in the leading edge of tumor, interact with CAFs in the TME, which promoted invasiveness and metastasis of OSCC.^61^ (C) Subpopulations and molecular signatures of immune cells revealed by scRNA‐seq. For example, both immunosuppressive checkpoints such as FOXP3 and CTLA4, and co‐stimulatory molecule genes such as CD28 and ICOS, were reported as marker genes of regulatory CD4+ T cells.^82^ OSCC, oral squamous cancer cell; CAFs, cancer‐associated fibroblasts; TME, Tumor Microenvironment; FOXP3, forkhead box P3; CTLA4, cytotoxic T lymphocyte‐associated protein 4; ICOS, Inducible T Cell Costimulator.

To the best of our knowledge, current research on oral cancer from scRNA‐seq perspective focus on squamous cell carcinoma. The illustration for key findings of oral squamous cell carcinoma through scRNA‐seq is shown in Figure 3.

### 4.2 Oral potential malignant disorders (OPMDs)

OPMDs have a statistically increased risk of progression into cancer. The most common OPMDs are oral submucous fibrosis (OSMF), leukoplakia, lichen planus, and erythroplakia.^95^ However, investigations into OPMDs were limited by the lack of means to analyze the small amount of cellular material.^96^ With the ability of assessing transcriptome heterogeneity and the requirement of low quantities of starting material, scRNA‐seq can solve these problems. The process of oral carcinogenesis is from the normal mucosa to precancerous lesions, and ultimately to cancer. The oral carcinogenesis can be induced by co‐stimulus of arecoline and 4‐nitroquinoline 1‐oxide (4‐NQO) in mouse model. It was shown by scRNA‐seq that genes of two cell subtypes which were likely to be essential during carcinogenesis, were associated with the MYC_targets_v1 pathway.^96^ These cell subtype markers can be applied to examine patients suffering from OPMD, to identify high‐risk populations, and used as a treatment target.^96^

As for OSMF, the presence of myofibroblasts (MFBs) and the constant α‐smooth muscle actin (α‐SMA) expression are regarded as landmark of deteriorating fibrosis and may change OSMF microenvironment, resulting in carcinogenesis.^97^ Subsets of myofibroblasts have been identified by scRNA‐seq in liver fibrosis,^98^ a premalignant lesion of the liver.^99^ It is reported that activated MFBs differentiated into various subpopulations, with markers such as of α‐SMA, collagens et al. S100 calcium binding protein A6 (S100A6) was considered as a general marker of activated MFB.^98^ It is promising that the MFB features identified in liver fibrosis supported by scRNA‐seq have implications for deciphering MFBs during the fibrosis of OSMF. It is worth mentioning that the cell subpopulations and transcriptome expressions of the MFBs in OSCC have been recorded by scRNA‐seq.^61^ Despite lack of related studies, it is believed that scRNA‐seq can illuminate the heterogeneity of MFBs in OSMF, which can be in comparison with scRNA‐seq data of MFBs in OSCC and thus demonstrate the difference between OSCC and OSMF in cell subpopulations and uncover potential underlying molecular mechanisms of how OSMF progresses into OSCC.

The scRNA‐seq has also been applied in premalignant diseases such as chronic atrophic gastritis and intestinal metaplasia,^100^ premalignant bronchial lesions,^101^ acinar metaplasia of pancreas^102^ etc., and these studies constructed a scRNA‐seq network underlying cellular and molecular characteristics of different premalignant lesions. Similarly, the scRNA‐seq can be applied in OPMD and establish an atlas of the biomarkers and cell subpopulations during the carcinogenesis. The scRNA‐seq was used to profile bronchial epithelial cells from smokers, leading to the characterization of metabolic alteration, tissue remodeling, the emergence of novel subpopulations, and markers related to premalignant lesions.^101^ Smoking is also a key risk factor of OMPD.^103^ The events emerging in bronchial epithelial cells caused by smoking inferred by scRNA‐seq can provide a reference for the research on OMPD resulting from tobacco, because a similar process may arise in the oral mucosal cells of OMPD caused by smoking which can be captured by scRNA‐seq. To the best of our knowledge, the studies of OPMD using scRNA‐seq are limited, but scRNA‐seq will undoubtedly bring new angles to study OPMD in future.

### 4.3 Oral and maxillofacial deformity

The orofacial clefting (OFC) can be divided into three sub‐phenotypes: clefting of the lip only (CL), clefting of both the lip and the palate (CLP), and clefting of the palate only (CP).^104^ Although a few risk genes associated with OFC have been identified, the molecular profiles and cellular lineages of the diseased malformation have not yet been integrated. The scRNA‐seq can be used to promote related research. The cell populations from endoderm, mesenchyme, and ectoderm participating in shaping and fusion of the facial process in the developing mouse face were identified by scRNA‐seq.^105^ The scRNA‐seq analysis revealed that the critical cell populations at the fusion site were within the periderm, and basal epithelial cells as well as adjacent mesenchyme. The unique transcriptome atlas of each cell population was revealed, especially the potential signals to manipulate their cell behavior. The scRNA‐seq data is essential to decode gene expression related to OFC in relevant cell clusters and understand how the fusion and patterning of facial prominences were influenced by genetic mutations or environmental elements.^105^ For example, SRY‐Box Transcription Factor 9 (SOX9) and TWIST1 were previously reported as the candidate risk genes causing CP,^106^ which were also detected by scRNA‐seq as marker genes of sub‐surface mesenchyme cell subpopulation outside the fusion zone of the facial prominences and a basal cell subpopulation at fusion zone, respectively.^105^ Using scRNA‐seq data combined with Whole‐Genome Sequencing (WGS) data, excessive loss‐of‐function de novo mutations (DNMs) were identified in genes enriched in craniofacial tissues, and markers related to OFC syndromes with autosomal dominant inheritance were also described.^104^

An epidermal commitment model was established by Soares et al.^107^ to induce the differentiation of human pluripotent stem cells (iPSCs) sampled from ectrodactyly, ectodermal dysplasia, and CL/P (EEC) syndrome patients with p63 mutations. Combined bulk RNA sequencing with scRNA‐seq was employed to trace cell trajectory throughout the epidermal differentiation. The iPSCs with p63 mutation showed differentiation defects during specification from the simple epithelium cells to the basal‐stratified epithelial fate. The scRNA‐seq‐based pseudotime trajectory analysis detected mesodermal activation that was relevant to the differentiation defects of EEC iPSCs.^107^ This research deciphered the potential mechanism of p63 mutation‐driven CL/P and provided an underlying treatment method for CL/P.

### 4.4 Infectious diseases

Infectious diseases often occur in the oral and maxillofacial region. The complex, and diverse interactions between the host cells and bacteria limit the depth of understanding the infectious diseases by bulk RNA sequencing.^108^ The scRNA‐seq, a technique powerful enough to identify intercellular heterogeneity and profile distinctive transcriptome reflecting each cell's biological characteristics,^109^ can remedy the shortages of bulk RNA sequencing. At present, combined with fluorescence labeling, scRNA‐seq analysis on host‐bacteria interaction can decipher heterogeneity of bacteria populations, as a mechanism which may help shape different host immune responses and remodeling of infected cells by bacteria through host genome plasticity.^109^, ^110^ The oral microbiota, the normal flora in oral cavity, consists of various species. In addition, microorganism is the culprit for most common oral diseases such as caries, gingivitis, and periodontitis.^111^ The host‐bacteria interactions revealed by scRNA‐seq could provide in‐depth knowledge of the oral bacterial infections and a novel perspective on the diagnosis and treatment of common oral bacterial infectious diseases.

Analysis on cell hierarchy and intercellular heterogeneity by scRNA‐seq has allowed profiling immune cell landscape^112^ and uncovering newly identified molecular mechanisms involved in regulating inflammation responses. First, the scRNA‐seq can be used for cluster analysis involved in periodontitis. After the single‐cell transcriptome profiles of healthy and periodontitis samples were compared, some novel cell subsets were identified. The research from Caetano et al. identified that one epithelial subtype driving the epithelial inflammation reaction increased, and a fibroblast‐like cluster had high expression in pro‐inflammatory genes such as amphiregulin (AREG).^39^ The scRNA‐seq study by Qian et al. reported that Major Histocompatibility Complex class II cell surface receptor HLA‐DR+ endothelial cells, CXCL13+ fibroblasts, and NLR Family Pyrin Domain Containing 3 (NLRP3) + macrophages were involved in the development of periodontitis.^113^ In addition, another study using scRNA‐seq revealed heterogeneity in macrophage populations between periodontitis‐affected and normal gingival tissues in individuals with and without type 2 diabetes.^114^ In the research from Chen et al., samples from healthy individuals and severe chronic periodontitis patients, as well as samples from severe chronic periodontitis patients prior to and following initial periodontal therapy, were analyzed by scRNA‐seq. Studying cell clusters in osteoimmunology microenvironments, TNF Receptor Superfamily Member 21 (TNFRSF21) + fibroblast subpopulations were identified as proinflammatory phenotype and CXCL12+ MSC‐like pericytes were recognized as contributing to pre‐osteoblasts during inflammation after periodontal therapy.^115^ Second, the gene expression in the scRNA‐seq dataset that was differentially regulated in periodontitis assisted in predicting cellular interactions with the aid of algorithms such as CellPhoneDB. It was found that the stromal cells were responsible for recruiting immune cells to the damage site of periodontitis, especially the neutrophil.^40^ The study by Qian et al. showed that increased intercellular communication arose between macrophages and T/B cells in periodontitis‐affected tissues.^113^ Signals that interacted with macrophages and other cell types were enriched in immune checkpoint pathways associated with exhaustion of T cells.^114^ Activation of Ephrin‐Eph signaling mediating crosstalk between endothelial cells and pre‐osteoblasts maybe responsible for pathological bone loss in periodontitis.^115^ Third, cell‐specific expression patterns of periodontitis susceptibility genes were identified. Most of the Mendelian disease genes related to periodontitis were expressed predominantly within the immune cells, except C1S and C1R which were expressed only in fibroblasts.^40^ In conclusion, the pathogenesis of periodontitis can be further understood by scRNA‐seq.

The advancement of scRNA‐seq promotes studying the heterogeneity of cellular response to viral infection.^116^ It is common that Human Immunodeficiency Virus (HIV) infection presents typical symptoms in oral cavity. The findings of several scRNA‐seq researches associated with immune response to HIV infection may contribute to the study on the pathogenesis of HIV‐infected oral lesions. The scRNA‐seq analysis on the peripheral blood mononuclear cells (PBMCs) from neonatal and adult monkeys immunized by HIV‐1 vaccine has demonstrated that T cells, B cells, natural killer (NK) cells, and monocytes from neonatal monkeys upregulated the expression of apoptosis regulator BCL2 and downregulated transcription of the immunosuppressive interleukin‐10 receptor alpha (IL10RA). In addition, immunized adult monkeys showed reduced frequencies of activated blood T follicular helper‐like (Tfh) cells in comparison with the neonatal ones. Therefore, compared to the adult ones, the immune profiles of neonatal monkeys were characterized by reduced immunosuppression and apoptosis.^117^ Moreover, Dendritic cells (DCs) isolated from HIV elite controllers were characterized by scRNA‐seq, and a subtype was endowed with increased antiviral response and antigen‐presentation. This DC subpopulation was enriched in HIV elite controllers in relative to chronic infectious patients and the healthy, which may possibly explain why the elite controllers had stronger immunological control.^118^ Furthermore, two major cell subpopulations with different susceptibility to HIV reactivation detected by scRNA‐seq showed different HIV transcript levels and distinct gene expression profiles, which may facilitate further advancement of “shock and kill” strategy to HIV infection.^116^ HIV suppresses and dysregulates the immune system of human tissues, particularly the mucosa. The impairment of HIV on mucosal barriers is progressive and irreversible.^119^ The immune atlas under HIV infection depicted by scRNA‐seq promotes understanding of the response of oral immune system to HIV invading and damage to innate immune components of the oral cavity by HIV, which will help develop therapeutic strategies and reduce HIV‐related oral complications.

The scRNA‐seq has been used to analyze the expression profiles of the host‐dependent molecules of Coronavirus Disease 2019 (COVID‐19) in cell populations from several human organs.^120^ Oral susceptible cells infected by COVID‐19 have been evaluated by scRNA‐seq. The severe acute respiratory syndrome coronavirus 2 (SARS‐CoV‐2), the pathogen of COVID‐19, invades human body through angiotensin‐converting enzyme 2 (ACE2) receptor,^121^ which was enriched in many human organs.^122^ Research with the combination of bulk RNA‐seq and scRNA‐seq revealed that the frequency of cells expressing ACE2 in alimentary canal organs was evidently higher than that in lung.^123^ The scRNA‐seq data from human minor salivary glands and gingival mucosa confirmed that the expression level of SARS‐CoV‐2 viral entry factor was higher in epithelium. More specifically, the ducts and acini of the salivary glands were involved as well as the mucosal upper‐basal cells. Furthermore, ACE2‐positive epithelial cells and SARS‐CoV‐2 RNA were found in the saliva sampled from patients suffering from COVID‐19.^59^ The invading mechanisms of SARS‐CoV‐2, confirmed by analysis on public scRNA‐seq datasets of human oral mucosa and immunohistochemistry, involved not only binding to ACE2 but also the fusion with cell membrane activated by Furin protease.^124^ In addition, a bioinformatic analysis on single‐cell transcriptomes from four oral mucosa tissues showed that the expression level of ACE2 was high in epithelial cells of tongue.^125^ In conclusion, the oral epithelial cells may be the susceptible cells; the oral cavity is likely to be a susceptible site for COVID‐19 infection and saliva plays an important role in viral transmission. The scRNA‐seq results help contribute to the possibility that the oral cavity can be the preventive and early diagnostic site for COVID‐19.

The scRNA‐seq probes into the etiopathogenesis and treatment of autoimmune diseases through the detection of transcriptome heterogeneity and important cell subpopulations.^1^ The scRNA‐seq was applied for detection on the diseased cell subpopulations and their expression profiles in primary Sjogren's Syndrome mouse salivary gland. The subsequent DEG and KEGG analysis demonstrated that affected acinar and ductal epithelial cell subpopulations may transition to a mixed epithelial/immune cell‐like state by aberrantly expressing molecules associated with immune cells.^126^ In addition, combined genome‐wide association studies (GWASs) and RNA‐seq data for Sjogren's syndrome with human parotid gland scRNA‐seq results, susceptibility genes in human parotid glands were identified.^58^

Systemic lupus erythematosus (SLE), a type of chronic and even life‐threatening autoimmune disease, is characterized by obscure pathogenesis and diverse manifestations. Oral discoid lesion is one of the most common SLE manifestations. Apart from petechial lesion, about half of SLE patients suffer from gingival bleeding such as desquamative gingivitis and chronic gingivitis.^127^ The scRNA‐seq analysis on renal and skin biopsies of SLE patients revealed that Type I interferon (IFN)‐sensitive markers in renal tubular cells and keratinocytes can assist in preventing the patients from suffering lupus nephritis. The markers associated with high sensitivity to IFN and fibrosis in renal tubular cells were relevant to therapeutic failure.^128^ Almost identical scRNA‐seq results have also been analyzed by Der et al.^129^ Keratinocyte of oral mucosal presented similar IFN‐response signatures, and thus biopsy of oral mucosal may help the diagnosis on lupus nephritis. In addition, the immune cell landscape in kidneys of SLE patients has been established by scRNA‐seq,^130^ which may provide a reference for the immune cell profile of oral site of SLE patients.

Rheumatoid arthritis (RA), another type of chronic autoimmune disease mainly affecting synovial joints, results in pain and restricted movement.^131^ It is reported that the temporomandibular joint (TMJ) is involved in RA.^132^ Utilizing scRNA‐seq, related studies on RA have uncovered the cell hierarchy in synovial tissue of RA and the corresponding unique gene expression profiles.^1^ Previously unidentified fibroblast subtypes and their locations in the synovium have been further clarified. Notch3 signaling has been proved to be critical to the pathological progress of perivascular and sublayer synovial fibroblasts in RA patients. Two subpopulations of fibroblasts with fibroblast activation protein α^+^ expression were found by scRNA‐seq and may help drive the progression of RA.^1^ A similar strategy can be applied to the synovial tissue of TMJ and may help explain the mechanism in autoimmune states of TMJ at single‐cell resolution.

### 4.5 Future directions

scRNA‐seq has brought revolutionary progress to the development of oral science. The main findings of researches on the application of scRNA‐seq in oral science are summarized in Table 2. Although the advancement of scRNA‐seq has revolutionized the perspective on our understanding of organisms, there are still lots of limitations in scRNA‐seq. The scRNA‐seq has the limitations on biopsy requisition. The volume of sample used by scRNA‐seq may be too small to reflect the whole damaged tissues. In addition, the tissue dissociation to obtain single cell cannot maintain the spatial data of the isolated samples.^1^ The samples collected for scRNA‐seq also have some limitations. For example, the current study collected normal and diseased tissues from periodontitis patients for scRNA‐seq. However, periodontitis is a systemic disease. Thus, sequencing samples from patients with periodontitis that are considered normal may not be representative of healthy periodontal tissue. Therefore, it is necessary to collect periodontal tissue from patients with completely healthy periodontium for scRNA‐seq.^111^ For most scRNA‐seq studies, samples were taken at a single time point. Samples should be taken at multiple time points to understand dynamic gene expression heterogeneity. In addition, non‐coding RNA acts as an important role in cancer, but scRNA‐seq including non‐coding RNA is still rare.^133^ Moreover, although the necessity of scRNA‐seq for microbes has been considered, it is halted by the difficulties including low mRNA transcripts in microbes, non‐polyadenylated mRNA of microbes, and bacterial cell‐wall‐based barrier, or cell‐membrane‐based barrier for cell lysis required for subsequent sequencing. Until recently, a scRNA‐seq method named microbial split‐pool ligation transcriptomics (microSPLiT) has been introduced to solve the difficulties in scRNA‐seq for microbes.^134^ More method development studies of scRNA‐seq should aim for enhanced sensitivity, reduced cost, higher throughput, and decreased technical noise,^13^ which will help extensive application of scRNA‐seq in basic research and clinical practice.

**TABLE 2:** Summary of main studies applying scRNA‐seq to oral science

Rank	Studies	Species	Biological samples	Number of subjects	Cells analyzed	Protocol used	Date published	Key findings
1	Sharir, A. et al.^22^	Mouse	Incisor	Controls Controls (*n* = 5) and 5FU‐treated (*n* = 5)	*n* = 8500	10× Genomics	Sep. 2019	Cellular hierarchies and mechanisms that underlie the homeostasis and repair of the mouse incisor
2	Chiba, Y. et al.^24^	Mouse	Incisor	–	*n* = 6260	10× Genomics	Sep. 2020	(1) Construction of entire cell populations in postnatal day 7 mice incisor (2) Identification of novel subpopulation of secretory‐stage ameloblasts
3	Krivanek, J. et al.^25^	Mouse & Human	Adult human healthy molar pulp and apical papilla; Incisor and molar pulp from mice	Mice for analysis of adult healthy mouse‐incisor (*n* = 39) and adult mouse molar pulps (*n* = 12); Human for analysis of healthy molar pulps (*n* = 7) and patients for analysis of apical papilla (*n* = 3)	Cells from mice (*n* = 31 164) and from human (*n* = 41 673)	Smart‐seq2 10× Genomics	Sep. 2020	(1) Identification of the terminal and transient cell states that enable self‐renewal and growth of mammalian teeth (2) Discovery on similarities and differences in tissue heterogeneity through comparisons of human and mouse teeth
4	Wen, Q. et al.^27^	Mouse	Mandibular first molar	Gli1‐Cre ^ERT2^;Runx2^fl/fl^ mice (*n* = 2) and Runx2^fl/fl^ mice (*n* = 2)	Cells from Gli1‐Cre ^ERT2^; Runx2 ^fl/fl^ mice (*n* = 4764) and Runx2 ^fl/fl^ (*n* = 4394)	10× Genomics	June 2021	Detection of the differential expression genes between Gli1‐Cre ^ERT2^; Runx2 ^fl/fl^ mice and Runx2 ^fl/fl^ mice
5	Shi, Y. et al.^30^	Human	Tooth germ tissue from third molars	Patients (*n* = 2)	*n* = 9855	–	Oct. 2021	Identification of cell subtypes and central signaling pathways from immature human tooth germ
6	Yin, W.et al.^31^	Human	Dental pulp from the first premolar	Patients (*n* = 2)	*n* = 12 114	–	Aug. 2021	(1) Construction of the dental pulp populations (2) The pulp cells communicated the most with other cell types, while T cells communicating the least
7	Pagella, P. et al.^32^	Human	Dental pulp and periodontal tissues	Third molars (*n* = 5)	Dental pulp cells (*n* = 32 378) and periodontal cells (*n* = 2883)	10× Genomics	May 2021	(1) Construction of cell cluster from human teeth (2) Similar molecular signatures were presented between dental pulp stem cells and periodontal stem cells, but microenvironment of them is different
8	Lee, S. et al.^33^	Human	Dental pulp and periodontal tissue from caries‐free premolar	Patients (*n* = 3)	hDPSCs (*n* = 8400) and hPDLSCs (*n* = 10 200)	–	Feb. 2022	Identification of expression profiles of human dental pulp stem cells and human periodontal ligament stem cells
9	Jones, K. B. et al.^38^	Mouse	Buccal mucosa	Mice (*n* = 10)	*n* = 16 572	10× Genomics	Nov. 2018	Construction of basal layer structure containing progenitor and post‐mitotic cells at various stages of maturation
10	Caetano, A. J. et al.^39^	Human	Buccal gingival margin	Controls (*n* = 2) and patients with periodontitis (*n* = 2)	*n* = 12 411	10× Genomics	Jan. 2021	(1) Construction of cell heterogeneity in human gingival tissue (2) Identification of changes in the transcriptome and cell populations between healthy and diseased patients' samples
11	Williams, D. W. et al.^40^	Human	Buccal and gingival mucosa	Health (*n* = 21) and periodontal disease (*n* = 8)	*n* = 88 000	10× Genomics	June 2021	(1) Construction of an scRNA‐seq atlas of human oral mucosa in the healthy controls and periodontitis patients (2) Stromal cell inflammatory profile is linked to neutrophil recruitment (3) Identification of cell‐specific expression patterns of periodontitis susceptibility genes
12	Lin, W. et al.^42^	Mouse	Mandibular alveolar bone tissue	Mice (*n* = 8)	*n* = 10 224	10× Genomics	Mar. 2021	(1) Identification of a more active immune microenvironment of alveolar bone (2) The macrophage subpopulation most actively interacts with MSCs subpopulation (3) Alveolar bone monocytes/macrophages express a higher level of Osm compared to long bone
13	Kwack, K. H. et al.^43^	Mouse	Femur and mandible bone	Mice (*n* = 2)	Cells from femur (*n* = 16140) and mandible (14 338) (*n* = 41 673)	10× Genomics	Sep. 2021	Discovery of heterogeneity of the myeloid lineage progenitor cell in alveolar (mandibular) bone versus long (femur) bone
14	Xu, J. et al.^50^	Mouse	Mandibular arch	Controls (*n* = 5) and mutant embryos (*n* = 5)	*n* = 10 586	10× Genomics	Jan. 2019	(1) Shh and Bmp4 signaling pathways are found to be activated in a complementary pattern along the oral‐aboral axis in mouse embryonic mandibular arch (2) Tissue‐specific inactivation of hedgehog signaling in neural crest derived mandibular mesenchyme led to expansion of BMP signaling activity to throughout the oral aboral axis of the distal mandibular arch and subsequently duplication of dentary bone in the oral side of the mandible at the expense of tongue formation
15	Song, E. C. et al.^52^	Mouse	Submandibular gland	–	*n* = 1013	10× Genomics	Sep. 2018	(1) Generation of a detailed map of the cell fate trajectories and branch points of the basal and myoepithelial cell populations of the mouse SMG during embryonic development and in adults (2) The p63+ cells contribute to and maintain all epithelial cell lineages during both embryogenesis and in the adult gland (3) The SMA+ myoepithelial cells only maintained the myoepithelial and ductal cell lineages in adults
16	Oyelakin, A. et al.^53^	Mouse	Parotid gland	–	*n* = 492	10× Genomics	Oct. 2019	(1) Identification of cellular heterogeneity in the parotid gland (2) Discovery on a novel cell subpopulation in equilibrium for commitment to the various cell lineages
17	Sekiguchi, R.^56^	Mouse	Submandibular and parotid glands	–	*n* = 14 441	10× Genomics	Oct. 2019	(1) Description of molecular signatures that define specific cellular landmarks for the bud initiation stage(2) Transcriptome data for embryonic parotid gland as compared with the submandibular gland with focusing on mesenchymal cell populations
18	Hauser, B. R. et al.^57^	Mouse	Submandibular gland	–	*n* = 24 722	10× Genomics	Nov. 2020	Identification of transcriptional profiles that revealed cellular heterogeneity during landmark developmental events of murine submandibular glands recruitment
19	Chen, M. et al.^58^	Human	Parotid gland	Patient (*n* = 1)	*n* = 16 052	10× Genomics	Feb. 2022	(1) Construction of an scRNA‐seq atlas of human parotid gland (2) The scRNA‐seq profiles of parotid and other digestive glands were compared (3) combined genome‐wide association studies (GWASs) and RNA‐seq data for Sjogren's syndrome with human parotid gland scRNA‐seq results, susceptibility genes in human parotid glands were identified
20	Huang, N. et al.^59^	Human	Gingival and minor salivary gland biopsy	Controls (*n* = 5) and patients (*n* = 5)	*n* = 8710	10× Genomics	Oct. 2020	SARS‐CoV‐2 viral entry factor is highly expressed in epithelia including the ducts and acini of the salivary glands and the suprabasal cells of the mucosae
21	Puram, S. V. et al.^61^	Human	Primary tumors and matching LN metastasis	Patients (*n* = 18)	*n* = 5902	SMART‐Seq2	Nov. 2017	(1) Reclassified HNSCC to 3 subtypes: basal‐mesenchymal, classical, atypical (2) p‐EMT program at tumor edge in proximity to CAFs (3) p‐EMT program recapitulated in LNs (4) p‐EMT program associated with regional metastasis and pathologic features
22	Chen, J. et al.^82^	Human	The OSCC tumor and paired adjacent normal tissues	Patients (*n* = 3)	*n* = 11 866	10× Genomics	Mar. 2021	(1) T‐cell subpopulations and their developmental trajectories within the tumors and the adjacent normal tissues (2) Exhausted CD8+ T cells and regulatory CD4+ T cells were enriched in OSCC tumors
23	Peng, Y. et al.^83^	Human	The OSCC tumor tissues	Patients (*n* = 6)	*n* = 51 728	10× Genomics	May 2021	(1) TCF1/TCF7 + T cells express high levels of TLS‐related genes and low levels of immune checkpoint molecules (2) TCF1/TCF7 + T cells were significantly associated with favorable outcomes
24	Cillo, A. R. et al.^85^	Human	Peripheral blood and HNSCC tumor specimens	HPV− HNSCC patients (*n* = 18) and HPV+ HNSCC patients (*n* = 8)	*n* = 131 224	10× Genomics	Jan. 2020	Transcriptional signature profiles of immune cells within tumors of HPV‐ and HPV+ HNSCC
25	Li, H. et al.^105^	Mouse	Lambdoidal junction area	Mouse embryos (*n* = 4)	*n* = 7893	10× Genomics	June 2019	(1) Identification of key cell populations at the fusion site exist within the periderm, basal epithelial cells and adjacent mesenchyme (2) Unique expression profiles of each population and the potential signals of integrating their behavior
26	Soares, E. et al.^107^	Human	Human embryonic stem cells (ESC), human induced pluripotent stem cells (iPSC), and human primary keratinocytes (KCs)	Patients (*n* = 2) and controls (*n* = 2)	*n* = 1250	STRT‐seq	Aug. 2019	(1) Identification of transcriptomic and genomic changes during differentiation of iPSCs derived from EEC patients caused by p63 mutations to epidermal cells(2) Consideration of mesodermal activation that was associated with the deviated commitment route of EEC iPSCs
27	Qian, S. J. et al.^113^	Human	Gingival tissues	Periodontitis patients (*n* = 2) and the health (*n* = 2)	Periodontitis patients (*n* = 10 161) and the health (*n* = 19806)	10× Genomics	Sep. 2019	Identification of HLA‐DR + endothelial cell, CXCL13+ fibroblast and NLRP3+ macrophages in the development of periodontitis
28	Agrafioti, P. et al.^114^	Human	Gingival tissue from periodontitis‐affected and healthy sites of patients	Periodontitis patients (*n* = 3)	*n* = 1109	10× Genomics	Jan. 2022	Definition of the heterogeneity of macrophages in gingival tissue from health and periodontitis patients
29	Chen, Y. et al.^115^	Human	Periodontal tissues	Controls (*n* = 4), patients with severe chronic periodontitis (*n* = 5), and patients with severe chronic periodontitis after initial periodontal therapy in 1 month (*n* = 3)	*n* = 51248	10× Genomics	Jan. 2022	Constructions of gene expression profiles, cell populations and intercellular crosstalk of the osteoimmunology microenvironment during periodontitis progression
30	Horeth, E. et al.^126^	Mouse	Submandibular gland	Primary Sjögren's Syndrome mice (*n* = 2) and controls (*n* = 2) molar pulps (*n* = 7) and patients for analysis of apical papilla (*n* = 3)	Primary Sjögren's Syndrome mice (*n* = 13846) and controls (*n* = 12000)	10× Genomics	Nov. 2021	Detection on the diseased cell subpopulations and their expression profiles in primary Sjogren's Syndrome mouse Submandibular gland

## AUTHOR CONTRIBUTIONS

J. Wu, Y. Ding and J. Wang contributed equally to the manuscript conception, drafting of this review, and wrote this paper; F. Lyu, Q. Tang, J. Song, Z. Luo, Q. Wan and X. Lan drafted and critically revised the manuscript; Z. Xu and L. Chen designed and revised the paper. All authors gave final approval and agreed to be accountable for all aspects of the work.

## CONFLICT OF INTEREST

The authors declare no competing interests.

## Data Availability

Data sharing is not applicable to this article as no new data were created or analyzed in this study.
